# Stereoselective recognition of morphine enantiomers by **μ**-opioid receptor

**DOI:** 10.1093/nsr/nwae029

**Published:** 2024-01-22

**Authors:** Yibo Wang, Van A Ngo, Xiaohui Wang

**Affiliations:** Laboratory of Chemical Biology, Changchun Institute of Applied Chemistry, Chinese Academy of Sciences, Changchun 130022, China; Advanced Computing for Life Sciences and Engineering Group, Science Engagement Section, National Center for Computational Sciences, Oak Ridge National Lab, Oak Ridge 37831, USA; Laboratory of Chemical Biology, Changchun Institute of Applied Chemistry, Chinese Academy of Sciences, Changchun 130022, China; School of Applied Chemistry and Engineering, University of Science and Technology of China, Hefei 230026, China; Beijing National Laboratory for Molecular Sciences, Beijing 100190, China

**Keywords:** stereoselective recognition, thermodynamics and kinetics, residence time, morphine enantiomers, μ-opioid receptor

## Abstract

Stereospecific recognition of chiral molecules plays a crucial role in biological systems. The μ-opioid receptor (MOR) exhibits binding affinity towards (−)-morphine, a well-established gold standard in pain management, while it shows minimal binding affinity for the (+)-morphine enantiomer, resulting in a lack of analgesic activity. Understanding how MOR stereoselectively recognizes morphine enantiomers has remained a puzzle in neuroscience and pharmacology for over half-a-century due to the lack of direct observation techniques. To unravel this mystery, we constructed the binding and unbinding processes of morphine enantiomers with MOR via molecular dynamics simulations to investigate the thermodynamics and kinetics governing MOR's stereoselective recognition of morphine enantiomers. Our findings reveal that the binding of (−)-morphine stabilizes MOR in its activated state, exhibiting a deep energy well and a prolonged residence time. In contrast, (+)-morphine fails to sustain the activation state of MOR. Furthermore, the results suggest that specific residues, namely D114^2.50^ and D147^3.32^, are deprotonated in the active state of MOR bound to (−)-morphine. This work highlights that the selectivity in molecular recognition goes beyond binding affinities, extending into the realm of residence time.

## Teaser

The μ-opioid receptor (MOR) exhibits distinct thermodynamic and kinetic recognition of morphine enantiomers, characterized by variations in the depth of energy wells and residence times. This study highlights the concept that molecular selectivity in molecular recognition extends beyond binding affinities and encompasses the temporal aspects of ligand-receptor interactions

## INTRODUCTION

**Figure 1.**
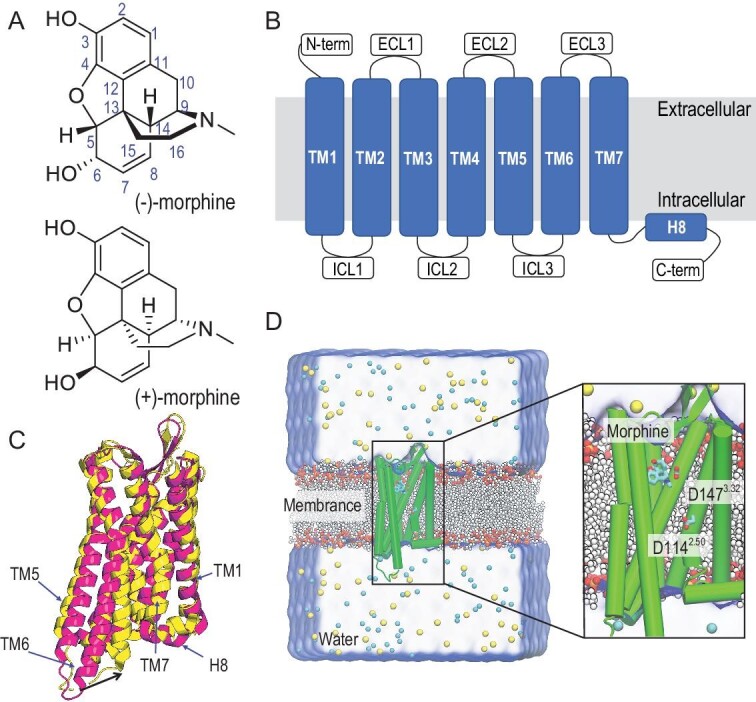
Structures of morphine and MOR. (A) Chemical structures of (−)-morphine showing its standard carbon numbering system and (+)-morphine. (B) Topology of MOR: seven transmembrane helices (TM1–TM7) with helix 8 (H8), connected with three extracellular (ECL1–ECL3) and three intracellular (ICL1–ICL3) loops. (C) Alignment of activated (PDB ID: 5C1M, magenta) and inactivated (PDB ID: 4DKL, yellow) MOR. The large displacement of TM6 is labeled via a black arrow. (D) A setup of a molecular dynamics (MD) simulation. A simulation system is composed of an MOR-morphine complex embedded in a 1-palmitoyl-2-oleoyl-sn-glycero-3-phosphocholine (POPC) and cholesterol bilayer with a mixing POPC : cholesterol ratio of 9 : 1 and a water solvent box. Na^+^ and Cl^−^ are shown as yellow and cyan spheres. Morphine and key residues D114^2.50^ and D147^3.32^ in MOR are shown as sticks. For clarity of the figure, only some lipid molecules and a part of the water box are shown and part of TM7 is removed.

Chirality is a fundamental property of molecules within living systems, where the phenomenon of stereoselectivity underlies numerous molecular recognitions in biological processes [1]. Chiral differences between enantiomers can lead to distinct interactions with biological receptors [2,3]. The stereoselective recognition of chiral enantiomers by receptors has attracted significant interest due to the universal importance of chirality in the realms of chemistry and medicine. Morphine, a powerful and longstanding analgesic drug used for treating severe pain, possesses five chiral centers (C5, C6, C9, C13, and C14 in Fig. 1A), which contribute to its pronounced stereoselectivity in analgesic effects [4]. The naturally occurring form of morphine, isolated from the dried latex of the opium poppy *Papaver somniferum*, is identified as the levorotatory isoform, (−)-morphine (Fig. 1A). (−)-Morphine exhibits potent analgesic effects by triggering μ-opioid receptors (MORs) as an agonist, while the synthetic enantiomer, (+)-morphine (Fig. 1A), demonstrates minimal activity in receptor binding assays and lacks analgesic activity [5].

MOR, a typical member of the G protein-coupled receptor (GPCR) family, is responsible for mediating the effects of potent analgesics. GPCRs, which are highly conserved and participate in numerous physiological processes, represent attractive targets for a significant proportion of drugs on the market [6,7]. Recent breakthroughs in structural biology have led to an enormous surge of high-resolution GPCR structures [8,9], offering opportunities to understand receptor stereoselectivity at the atomic level. MOR shares the common GPCR structural core, comprising seven transmembrane helices (TM1–7 in Fig. 1B) and a helix (H8) nearly parallel to the membrane surface, connected by three extracellular loops (ECL1–3) and three intracellular loops (ICL1–3). The N-terminus of the GPCR is positioned on the extracellular side, while the C-terminus resides intracellularly. Activation of MOR leads to conformational changes at the intracellular ends of transmembrane helices, with TM6 undergoing a substantial outward movement to recruit downstream signaling effectors (Fig. 1C) [8]. Since the powerful analgesic and addictive properties of opiate alkaloids are mediated by MOR as agonists [10], the activated structure has been extensively studied through *in silico* simulations [11–16]. Nevertheless, the mechanism underlying MOR's stereoselective recognition of morphine enantiomers, a puzzle in neuroscience and pharmacology existing since the identification of MOR's stereoselectivity towards morphine enantiomers in 1977 [5], remains elusive.

Agonists typically serve two roles in receptor interactions. For agonist-dependent signaling or activation, the agonist binds to the receptor and activates it, thereby triggering signaling events. However, recent findings have revealed that the activation mechanisms of GPCR, including MOR, go beyond agonist-dependent signaling; they also manifest agonist-independent basal or constitutive activity [17–19]. Although MORs typically assume inactive conformations without an agonist, some can spontaneously adopt an active conformation. This creates a dynamic equilibrium between the active and inactive states, with agonist binding serving to shift this equilibrium by primarily stabilizing the active state [19]. In this study, molecular dynamics (MD) simulations were performed to compare the efficacy of morphine enantiomers in stabilizing the active state of MOR and the binding process, aiming to elucidate MOR's stereoselective recognition.

The description of receptor-ligand binding can be summarized by two primary factors: thermodynamics and kinetics [20]. Thus, MOR's stereoselectivity toward morphine enantiomers can be quantified in terms of two aspects: thermodynamics, as measured by binding energy; and kinetics, as reflected by the duration of ligand efficacy within the target, known as residence time [21,22]. In this work, the stereoselectivity of MOR toward morphine enantiomers was addressed by comparing the binding energies and residence times of the enantiomers with MOR at different protonation states. (−)-Morphine exhibited the deepest energy well (−18.39 kcal/mol) in deprotonated MOR at residues D114^2.50^ and D147^3.32^ (superscripts refer to the Ballesteros-Weinstein generic numbering scheme [23], see Fig. 1D), accompanied by the longest residence time (0.30 ± 0.02 s). The activation of MOR depended on both the agonist and specific protonation states of key residues. (−)-Morphine effectively stabilize the activated state of MOR with deprotonated D114^2.50^ and D147^3.32^ residues. However, it failed to maintain the activated state when D114^2.50^ was protonated and D147^3.32^ was deprotonated. Furthermore, MOR bound to (+)-morphine tended to lose its active conformation regardless of the protonation states of D114^2.50^ and D147^3.32^. Considering that (+)-morphine displayed shallow energy wells with short residence times (ranging from microseconds to milliseconds) upon binding to MOR and was unable to stabilize the activated state, it is not surprising that (+)-morphine lacks analgesic activity. To our knowledge, this study is the first to dissect the thermodynamics and kinetics of MOR's stereoselective recognition of morphine enantiomers.

## RESULTS

The p*K*_a_ value of D114^2.50^ in the MOR/morphine complex was predicted to be approximately 6.7 using PROPKA3 [24]. This prediction suggests that the deprotonated or charged state of D114^2.50^ is predominantly present within the complex. However, previous studies have proposed that in certain GPCRs, the residue D^2.50^ may be protonated in the active state and deprotonated in the inactive state [25–28]. Consequently, the neutral state of D114^2.50^ in the MOR complex was also considered during the simulations. D147^3.32^ was initially assigned as the charged state to establish a salt bridge with the protonated ligand. It is worth noting that even in its neutral state, D147^3.32^ can still form hydrogen bonds with morphine. Furthermore, given the dynamic nature of protonation states, the MOR complex with protonated D147^3.32^ was also investigated in this study. Considering the chiral states of morphine, a total of eight systems were constructed. A summary of these simulations is provided in Table S1.

The root-mean-square deviations (RMSDs) of the MOR backbone and the root-mean-square fluctuations (RMSFs) for the Cα atoms of MOR were calculated first. The RMSDs in Fig. S1 indicated that all simulation systems reached stable states within 100 ns of simulation. The RMSFs offer crucial insights into the fluctuations of our simulation systems. As shown in Fig. S2, large fluctuations only occurred in the loop regions and termini. During the preparation of this manuscript, the cryo-EM structure of (−)-morphine-bound human MOR was resolved [29]. The structural alignment was performed to evaluate the accuracy of our model, and the results are presented in Fig. S3. Impressively, our equilibrated docking structure of (−)-morphine-bound MOR demonstrated remarkable similarity to the conformation observed in the cryo-EM structure, emphasizing the accuracy of the computational approach.

**Figure 2.**
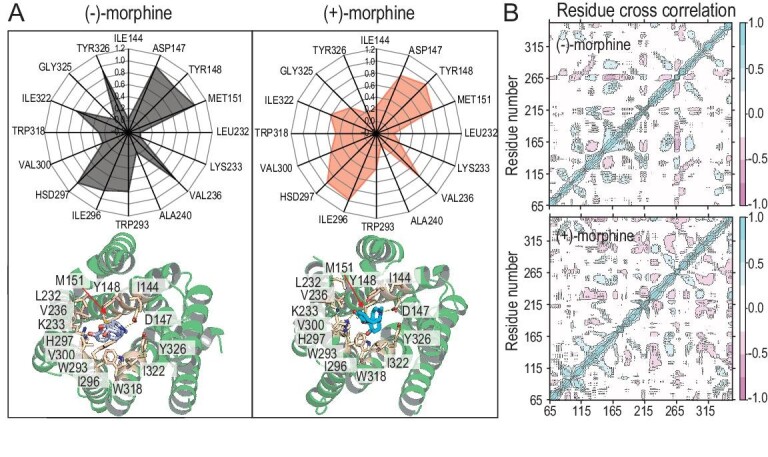
Interactions of morphine with MOR and MOR behaviors when D114^2.50^ and D147^3.32^ were deprotonated during equilibration. (A) Molecular interaction fingerprints between D114^2.50^- and D147^3.32^-deprotonated MOR and (−)-morphine/(+)-morphine based on the last 50 ns simulations. The numbers 0–1.0 in the radar chart indicate the probability of interactions between morphine and a certain residue derived from 5000 snapshots extracted from the last 50 ns simulations. The side chains of key residues involved in the interaction with morphine are displayed. (B) Dynamic cross-correlation analysis of Cα atoms in MOR during the last 50 ns trajectory. The color bar indicates the degrees of correlation and anti-correlation.

The interactions between morphine and MOR, as well as the behavior of MOR during equilibration, are shown in Fig. 2 and Figs S4–S7. In the case of MOR with deprotonated D114^2.50^ and D147^3.32^, the interaction fingerprints of (−)-morphine-MOR revealed the involvement of nine residues (D147, Y148, M151, V236, W293, I296, H297, I322, and Y326) in three types of interactions: salt bridge, hydrogen bonds, and hydrophobic interactions (Fig. 2A). However, for (+)-morphine, only six residues (D147, Y148, M151, V236, I296, and H297) exhibited frequent interactions with the ligand. Dynamic cross-correlation analysis of (−)-morphine with deprotonated MOR at D114^2.50^ and D147^3.32^ (Fig. 2B) indicated an anti-correlated conformational motion between TM3 (residues 149–199) and residues 260–270 in ICL3. This anti-correlation was less pronounced in the case of (+)-morphine-bound deprotonated MOR at D114^2.50^ and D147^3.32^ (Fig. 2B). Additionally, (−)-morphine-bound MOR exhibited a similar number of nodes compared to the (+)-morphine-bound MOR (Fig. S4A, C). The dynamic communication between D114^2.50^ and D147^3.32^ in (−)-morphine-bound MOR occurred via N150 (Fig. S4B), which differs from the (+)-morphine-bound MOR where communication occurred via N150 and M151 (Fig. S4D). Regardless of the protonation state of D114^2.50^ and D147^3.32^, (−)-morphine and (+)-morphine displayed distinct interaction fingerprints with their respective MORs, and the MORs exhibited unique dynamic behaviors influenced by their ligands (Figs S5–S7).

To ascertain the thermodynamic and kinetic information of morphine binding to MOR, a series of Markov state models were built based on the reaction coordinate with increasing lag times. Figure S8 demonstrates that the dynamics of the morphine-bound MOR systems converged when the lag time reached or exceeded 160 steps (1.6 ns). Consequently, a lag time of 160 steps (1.6 ns) was selected to construct a Markov state model for subsequent thermodynamic and kinetic analyses.

### Thermodynamics of morphine binding to MOR under varying protonation states of D114^2.50^ and D147^3.32^

**Figure 3.**
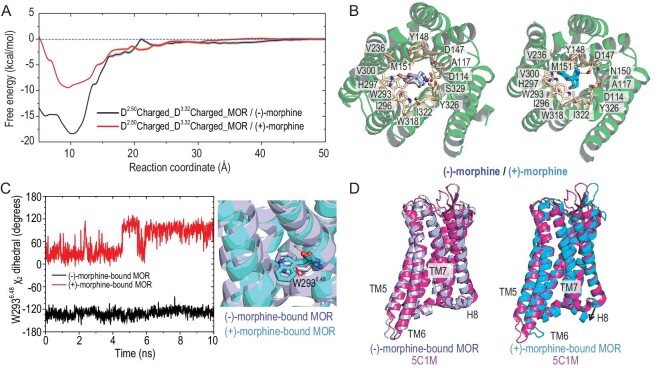
Thermodynamic analysis of morphine enantiomers binding to D114^2.50^- and D147^3.32^-deprotonated MOR. (A) Free energy profiles of (−)-morphine and (+)-morphine binding to D114^2.50^- and D147^3.32^-deprotonated MOR. Errors in the free energy calculations are determined using a bootstrapping procedure. (B) Binding pocket residues of (−)-morphine and (+)-morphine in D114^2.50^- and D147^3.32^-deprotonated MOR, visualized from the extracellular side. (C) W293^6.48^ flipping into the binding site to establish hydrophobic interactions and stabilize (−)-morphine in the activated MOR. The dihedral of W293^6.48^ is monitored during the simulations. (D) Alignment of (−)-morphine-bound MOR and (+)-morphine-bound MOR with the activated crystal structure of MOR (PDB ID: 5C1M), highlighting the significant conformational changes compared to the activated state, indicated by arrows.

The thermodynamics of morphine binding to MOR were investigated under different protonation states of critical residues, specifically D114^2.50^ and D147^3.32^. The free energy profiles, calculated using dTRAM, provided crucial insights into the energetics of these interactions. As shown in Fig. 3A, (−)-morphine exhibited a deep energy well with −18.39 kcal/mol at a reaction coordinate, *z* = 10 Å, when both D114^2.50^ and D147^3.32^ were in their deprotonated states. In contrast, the global minimum energy profile for (+)-morphine was −9.47 kcal/mol at *z* = −9.5 Å. This observation highlighted the preferential energetics of (−)-morphine over (+)-morphine in MOR when both D114^2.50^ and D147^3.32^ were deprotonated. Intriguingly, the two morphine enantiomers adopted distinct conformations within their respective global minima. For (+)-morphine, the tertiary amine nitrogen established a salt bridge with the charged D147^3.32^, and the phenol group engaged in a hydrogen bond with H297^6.52^ (Fig. 3B). Conversely, (−)-morphine preferred a conformation akin to morphine and its derivative BU72 in the active crystal structures of MOR [29,30]. In this orientation, the tertiary amine nitrogen of (−)-morphine faced to D114^2.50^, creating stronger electrostatic interactions with a shorter distance compared to (+)-morphine. The phenol group continued to interact with H297^6.52^, while additional interactions formed. These included D147^3.32^, A117^2.49^, Y326^7.43^, and the tertiary amine nitrogen of (−)-morphine forming a network, mediated by a water molecule (Fig. 3B). Furthermore, W293^6.48^ turned into the binding site to stabilize (−)-morphine through hydrophobic interactions (Fig. 3C). Importantly, when compared to the activated crystal structure (PDB ID: 5C1M), (−)-morphine was found to stabilize MOR with deprotonated D114^2.50^ and D147^3.32^ in the activated state (Fig. 3D). In contrast, the binding of (+)-morphine resulted in the displacement of H8, driving MOR towards the intermediate state [31], indicating that (+)-morphine could not stabilize MOR in the activation state. Combining the computational results with the well-established experimental knowledge that (−)-morphine potently stimulates MORs to produce analgesic effects, D114^2.50^ is suggested to be deprotonated in the (−)-morphine-bound active MOR.

In scenarios where D114^2.50^ was deprotonated and D147^3.32^ was protonated, an interesting shift was observed in binding preferences. Specifically, (+)-morphine became approximately 3 kcal/mol more energetically favorable for binding to MOR than (−)-morphine (Fig. S9A). In these conditions, neither (−)-morphine nor (+)-morphine engaged in hydrogen bonding interactions with D147^3.32^, as the protonated state of D147^3.32^ led it to point away from the binding site (Fig. S9B). Nevertheless, the phenol group of (+)-morphine still formed a hydrogen bond with H297^6.52^. Importantly, while the extracellular ends of TM6 and TM7 underwent relocation in the (−)-morphine-bound MOR, the intracellular ends of TM5 and TM6, along with the ICL3 loop, remained relatively unchanged (Fig. S9C). This indicated that MOR, in the presence of (−)-morphine, maintained its activated conformation. Conversely, binding of (+)-morphine led to an inward movement of the intracellular ends of TM5 and TM6, along with the ICL3 loop, resulting in the inactivation of the receptor.

When D114^2.50^ was protonated and D147^3.32^ was deprotonated, a significant preference for (+)-morphine, which was approximately 10 kcal/mol more energetically favorable for MOR binding than (−)-morphine, was revealed (Fig. S10A). Both morphine enantiomers formed stable salt bridges with D147^3.32^ and hydrogen bonds with H297^6.52^ (Fig. S10B). Notably, the presence of (+)-morphine induced an inward displacement of TM6, a clear indicator of MOR inactivation (Fig. S10C). Meanwhile, (−)-morphine adopted a similar orientation to that observed in the MOR state with deprotonated D114^2.50^ and D147^3.32^, where the oxygen in the oxide bridge interacted with Y148^3.33^ via a water bridge. However, the binding energy well was too shallow to maintain MOR activation, as TM6 of MOR also shifted inward (Fig. S10C). Regarding the well-established experimental evidence that (+)-morphine has minimal affinity for MOR, it is unlikely that the D114^2.50^-protonated and D147^3.32^-deprotonated state of MOR occurs in reality.

When both D114^2.50^ and D147^3.32^ were protonated, (−)-morphine became slightly less energetically favorable for MOR binding, approximately 1.7 kcal/mol less favorable than (+)-morphine (Fig. S11A). Both enantiomers established hydrogen bonds with H297^6.52^. However, due to the protonated D147^3.32^ pointing away, (−)-morphine was unable to interact with this residue. Instead, the tertiary amine nitrogen of (−)-morphine formed interactions with Y326^7.43^ via a water bridge (Fig. S11B). Protonated D147^3.32^ remained close to the tertiary amine nitrogen of (+)-morphine, allowing interactions to occur. (−)-Morphine, under these conditions, stabilized MOR in the activated state with a slight shift of TM5, while TM6 of (+)-morphine-bound MOR displaced an inward shift (Fig. S11C). It is worth noting that (−)-morphine had to overcome a barrier of approximately 1.8 kcal/mol to enter the binding site (Fig. S11A), rendering the binding process with D114^2.50^-protonated and D147^3.32^-protonated MOR kinetically unfavorable.

Taken together, these *in silico* simulations imply the pivotal role of the protonation states of key residues D114^2.50^ and D147^3.32^ in stabilizing the activated state of MOR by (−)-morphine. The binding of (+)-morphine was associated with a loss of the active conformation. It is important to acknowledge that the protonation states of MOR's key residues are dynamic in nature [25–28]. Most likely, the binding process represents a combination of various protonation states with a specific partition function. Since the side chain environment of aspartic acids keeps changing during the ligand binding or unbinding process, it is difficult to determine the partition function. Given the experimental facts that (−)-morphine effectively binds and activates MOR while (+)-morphine exhibits minimal MOR affinity, it is highly probable that D114^2.50^ and D147^3.32^ are deprotonated in the active MOR state.

### Kinetics of morphine binding to MOR under varying protonation states of D114^2.50^ and D147^3.32^

The binding kinetics between a drug molecule and its biological target are described by the association rate constant *k_on_* and the dissociation rate constant *k_off_* (or drug residence time *t_out_*, defined as 1/*k_off_*). In addition to the binding affinity, drug residence time has been increasingly considered a crucial factor for dictating much of the drug pharmacological activity [21,22]. In this study, Markov state models were employed to elucidate the kinetics of morphine enantiomers binding to MOR under varying protonation states of D114^2.50^ and D147^3.32^. Microstates in the Markov model for each system were simplified into two metastable states: the bound and unbound states.

**Figure 4.**
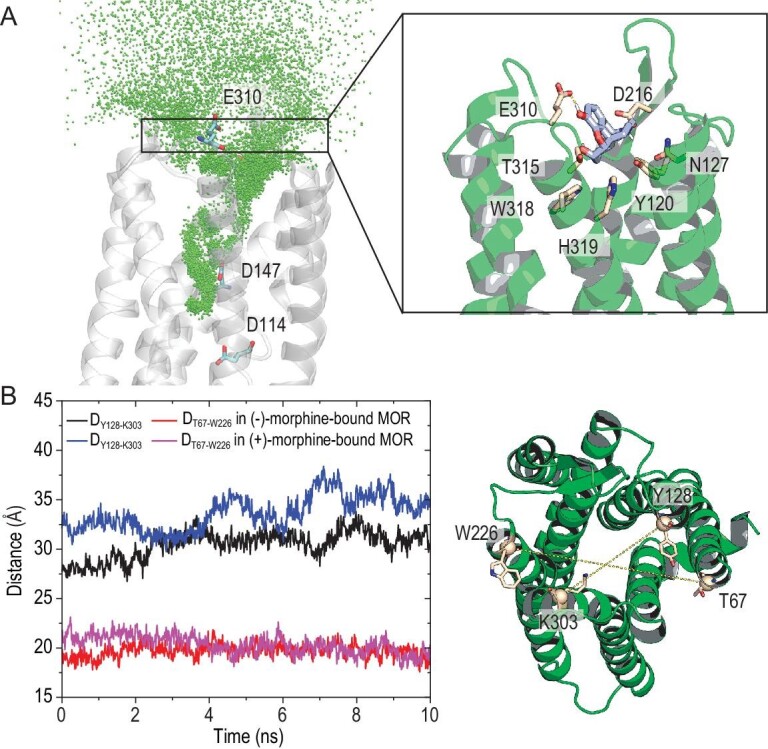
Transition state of (−)-morphine binding to D114^2.50^- and D147^3.32^-deprotonated MOR. (A) The binding and unbinding pathway of (−)-morphine with D114^2.50^- and D147^3.32^-deprotonated MOR, represented as the superposition of the center of mass of (−)-morphine over a time period of 2.02 μs (unbiased simulations). Key residues surrounding (−)-morphine in the transition state are shown in sticks. (B) Distance of Cα atoms of Y128-K303 and T67-W226 in (−)-morphine-bound MOR (*z* = 22.5 Å) and (+)-morphine-bound MOR (*z* = 19.5 Å).

For (−)-morphine binding to D114^2.50^-protonated and D147^3.32^-protonated MOR, the transition time of 99.07 ± 5.37 ns was estimated at a concentration of 0.70 mM, which was comparable to that of (+)-morphine at 63.79 ± 2.12 ns (Table 1). However, the residence time of (−)-morphine (0.30 ± 0.02 s) exceeded that of (+)-morphine (37.36 ± 4.16 μs) by a remarkable factor of 8000. The transition state of (−)-morphine release process was located at *z* = 22.5 Å. At this location, (−)-morphine was placed between D216^ECL2^ and E310^ECL3^, exerting force to push ECL2 and ECL3 away (Fig. 4A). These extracellular loops are known to act as a ‘gate’, influencing ligand access to the binding pocket and its release, and their behavior is linked with MOR activation [32,33]. In contrast, the transition state for (+)-morphine binding (*z* = 19.5 Å) witnessed the outward movement of the extracellular ends of the MOR helices compared to the (−)-morphine-bound MOR. This shift created a larger space for (+)-morphine to access (Fig. 4B), potentially explaining the slightly faster binding of (+)-morphine. Nonetheless, no specific interactions between (+)-morphine and MOR were observed in the transition state.

**Table 1.** Kinetic parameters for (−)-morphine and (+)-morphine interacting with MOR.

		Binding time *t_in_* (ns)	Residence time *t_out_*	Association rate constant *k_on_* [1/(ns mM)]
**D^2.50^Charged_D^3.32^Charged_MOR**				
	(−)-morphine	99.07 ± 5.37	0.30 ± 0.02 s	0.01434 ± 0.00067
	(+)-morphine	63.79 ± 2.12	37.36 ± 4.16 μs	0.02227 ± 0.00109
**D^2.50^Charged_D^3.32^Neutral_MOR**				
	(−)-morphine	336.32 ± 16.26	4.36 ± 0.61 μs	0.00422 ± 0.00022
	(+)-morphine	326.46 ± 46.44	49.87 ± 11.87 μs	0.00435 ± 0.00030
**D^2.50^Neutral_D^3.32^Charged_MOR**				
	(−)-morphine	523.36 ± 38.43	1.90 ± 0.22 μs	0.00271 ± 0.00013
	(+)-morphine	46.82 ± 1.21	4.57 ± 0.65 ms	0.03035 ± 0.00076
**D^2.50^Neutral_D^3.32^Neutral_MOR**				
	(−)-morphine	1153.46 ± 68.69	52.72 ± 3.42 μs	0.001231 ± 0.00010
	(+)-morphine	198.76 ± 13.02	59.40 ± 6.05 μs	0.00715 ± 0.00058

**Figure 5.**
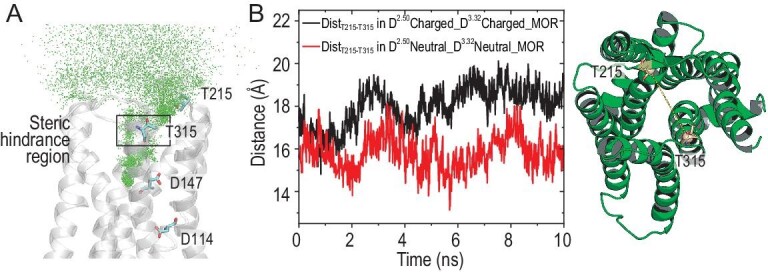
Transition state of (−)-morphine binding to D114^2.50^- and D147^3.32^-protonated MOR. (A) The binding and unbinding pathway of (−)-morphine with D114^2.50^- and D147^3.32^-protonated MOR, represented as the superposition of the center of mass of (−)-morphine over a time period of 2.02 μs (unbiased simulations). The region of steric hindrance is labeled to indicate potential obstacles during binding. (B) Distance between the Cα atoms of T215 and T315 in morphine-bound MOR. Black represents D114^2.50^- and D147^3.32^-deprotonated MOR (*z* = 22.5 Å), while red represents D114^2.50^- and D147^3.32^-protonated MOR (*z* = 18.5 Å).

Binding of morphine enantiomers to D114^2.50^-deprotonated and D147^3.32^-protonated MOR required over 300 ns at a concentration of 0.70 mM. The residence times were 4.36 ± 0.61 μs for (−)-morphine and 49.87 ± 11.87 μs for (+)-morphine. The protonation of D147^3.32^ exerted a dramatic influence on the binding kinetics of (−)-morphine. When D114^2.50^ was protonated and D147^3.32^ was deprotonated, the residence time of (+)-morphine (4.57 ± 0.65 ms) was 2400 times longer than that of (−)-morphine (1.90 ± 0.22 μs). Apparently, in this situation, MOR exhibited a pronounced kinetic preference for (+)-morphine. Finally, when both D114^2.50^ and D147^3.32^ were protonated, (−)-morphine had to overcome a small energetic barrier at *z* = 19 Å to access MOR, slowing the binding process. The protonation of MOR in this state led to a reduction in the size of the extracellular entrance, resulting in steric hindrance that further delayed the binding of (−)-morphine (Fig. 5).

The analysis of kinetics sheds light on the chiral recognition capability of MOR, as reflected by the residence time. Notably, (−)-morphine demonstrated the longest residence time in the context of D114^2.50^- and D147^3.32^-deprotonated MOR. Conversely, the residence time of (+)-morphine with MOR under any protonated state investigated was significantly shorter than that of (−)-morphine interacting with the D114^2.50^- and D147^3.32^-deprotonated MOR. These thermodynamic and kinetic results are in line with experimental evidence demonstrating that (−)-morphine functions as an MOR agonist while (+)-morphine exhibits minimal affinity for MOR.

## DISCUSSION

Chirality is a fundamental molecular characteristic in living systems. Our study delved into the intricate mechanism underlying MOR's stereoselective recognition of morphine enantiomers. To unravel this longstanding puzzle, we conducted extensive, large-scale enhanced and unbiased samplings of morphine binding to MOR. Our findings, rooted in analyses of binding energy profiles and residence times, demonstrate that only (−)-morphine has the capability to stabilize MOR in an activated state with a prolonged residence time. In contrast, (+)-morphine fails to elicit the same response from the receptor. Moreover, the protonation states of key residues emerge as critical determinants in MOR's capacity to selectively recognize morphine enantiomers. When both D114^2.50^ and D147^3.32^ were deprotonated, (−)-morphine exhibited a clear preference for binding to MOR over (+)-morphine, maintaining MOR in the active state. However, when D114^2.50^ and D147^3.32^ were in different protonation states, (+)-morphine showed a preference for MOR binding over (−)-morphine. Yet, none of the complexes with (+)-morphine maintained the active state. This study demonstrates that (−)-morphine may serve a role similar to certain agonists known to enhance MOR activity by boosting the proportion of its fully activated conformation in the conformational equilibrium [19]. Nevertheless, there is a crucial need to delve deeper into the activation process of MOR by (−)-morphine, particularly in light of the clear lack of such activation by (+)-morphine. This exploration is vital for both neuroscience and pharmacology. It promises to unravel the specific structural and dynamic changes caused by the interaction of (−)-morphine with MOR, thereby shedding light on the distinctions in its binding patterns with MOR in comparison to (+)-morphine. However, it is crucial to note that transitions between the active and inactive states of MOR are estimated to occur within the millisecond range [34], making them challenging to observe within currently feasible simulation time frames even by enhanced sampling methods. This is not surprising considering that the activation of MOR is a complex and intricate process, involving proton transitions, ion modulation, G protein coupling, *etc*. This study concentrated on how MOR recognizes and binds to morphine in a stereoselective manner. Future studies exploring the activation process of (−)-morphine, as opposed to the inert behavior of (+)-morphine, could deepen our understanding of opioid receptor function.

In summary, this work illuminates the enigmatic question of why MOR selectively recognizes (−)-morphine over (+)-morphine at the atomic level. The selectivity in molecular recognition extends beyond binding affinities. The residence time of the ligand within the receptor also plays a pivotal role in determining the functional outcome. This endeavor may offer insights that can inform the development of safer and more efficacious analgesic agents.

## Supplementary Material

nwae029_Supplemental_File

## Data Availability

The data are available on request from the authors.

## METHODS

Detailed methods are available in the Supplementary data.
