# Supplementary material for: Stereoselective recognition of morphine enantiomers by μ-opioid receptor
Source: Natl Sci Rev. 2024 Jan 22;11(3):nwae029. doi: 10.1093/nsr/nwae029 (PMC10896590; doi:10.1093/nsr/nwae029)
Supplement: nwae029_Supplemental_File [file nwae029_supplemental_file.docx]

Supplementary Data

Methods

### Systems setup

The active μ-opioid receptor (MOR) structure (PDB ID: 5C1M) was obtained from a previous study[1]. The initial conformation of (-)-morphine was accomplished by docking it to this MOR structure via AutoDock Vina 1.1.2[2]. For (+)-morphine, it was modified manually based on the (-)-morphine with MOR. The morphine tertiary amine nitrogen was protonated, corresponding to the dominant protonation state at pH 7.0. To explore the influence of protonation states on MOR's enantioselective recognition, different protonation states to the critical residues D114^2.50^ and D147^3.32^ were assigned. Other titratable residues retained their dominant protonation state at pH 7.0. The MOR models with morphine enantiomers were embedded in a POPC and cholesterol bilayer with a mixing POPC:cholesterol≈9:1 ratio (**Figure 1D**). The membrane and protein were then neutralized and solvated with 150 mM NaCl and explicit TIP3P water. The entire simulation system contained approximately 223,000 atoms within a periodic boundary box measuring of 123×123×157 Å^3^ and was assembled using the CHARMM-GUI webserver[3]. The CHARMM36m force field was applied for the modeling of proteins, lipids and ions[4, 5]. The parameters for morphine enantiomers were generated by the GAAMP webserver[6].

All molecular dynamics (MD) simulations were performed using the NAMD2.12 software package[7]. Each system was simulated in an NPT ensemble with the temperature and pressure maintained at 310.15 K and 1 atm using the Nosé-Hoover thermostat[8] and Langevin piston method[9], respectively. The particle-mesh Ewald algorithm was applied to treat the long-range electrostatic interactions, and the nonbonded interactions were switched off from 10 to 12 Å[10].

After a multistep equilibration with gradually decreasing harmonic constraints on lipid and protein heavy atoms according to the CHARMM-GUI membrane builder equilibration protocol, an additional 100 ns unconstrained equilibration run was carried out to equilibrate the whole system. The last snapshot of each equilibration run was used as the initial configuration for subsequent simulations. For visualization purpose, PyMol[11] and VMD[12] were employed to analyze and visualize the structures. Three parallel simulations for each system were conducted to make sure the results are repeatable.

### Trajectory analysis

Based on the simulation trajectories, the following properties were analyzed:

**MOR-morphine interaction fingerprint analysis.** The molecular interaction fingerprints between MOR and morphine were calculated using ProLIF[13] with the default parameters, focusing on 5000 snapshots extracted from the final 50 ns simulations. In each frame, only residues within a 6 Å radius of the ligand’s center of mass were analyzed. Interactions, including hydrophobic, aromatic, h-bond donor, h-bond acceptor, positively ionizable, and negatively ionizable interactions of the atoms of proteins and ligands were evaluated. The results, in the form of interaction fingerprints, for all 5000 snapshots were graphically represented through radar charts.

**Dynamic cross-correlation network.** The evaluation of whether the fluctuations in one residue correlated with those in another was accomplished by calculating cross-correlation coefficients. Bio3D[14] was used for the calculation of dynamic cross-correlation networks for morphine-bound MOR in various protonation states.

**Residue communication network analysis.** This analysis involved the implementation of a community clustering procedure to divide the complete cross-correlation network into localized substructures or communities with Bio3D[14-16]. The network nodes represent a cluster of residues, which are connected through edges weighted by the correlation values between the two clusters. Optimal and suboptimal path calculations with WISP software[17] were also performed on each network to identify residues involved in the dynamic coupling of residues D114^2.50^ and D147^3.32^. The resulting path ensemble illustrated the various pathways through which dynamic communication could flow between D114^2.50^ and D147^3.32^. For each network, 500 paths, a number that was found to yield converged results in all cases[15], were collected for the D114^2.50^ and D147^3.32^ pair. Residues with a high node degeneracy (≥ 30 paths) in any network were specified as “on-path” residues.

### Umbrella sampling and unbiased simulations

To explore the binding process of morphine in MOR, umbrella Sampling (US) simulations were performed along the *z*-axis, parallel to the normal vector of the membrane surface. Harmonic biasing potentials were applied with a force constant of 10 kcal/(mol·Å^2^). The reaction coordinate was defined as the distance along the *z*-axis between the center of mass of the ligand and the center of mass of the Cα atom of residue D114^2.50^. For each system, sampling windows were spaced every 0.5 Å from 5 Å to 55 Å, resulting in 101 windows for each US simulation to cover the entire binding path. A flat-bottom cylindrical constraint with a radius of 15 Å was applied to avoid insufficient sampling of morphine in the bulk solvent. To prevent the drift of MOR within the membrane, a harmonic potential was applied to the head groups of POPC lipids with a force constant of 5 kcal/(mol·Å^2^). Each of the US window was run for 11 ns, with the last 10 ns of data being extracted for subsequent analysis.

To assess the kinetic information of morphine binding to MOR, a series of unbiased simulations were performed for 20 ns with the last frame of each umbrella sampling window as the initial structure. The same simulation settings as the biased simulations were applied except all restraint potentials on the ligands were removed.

### Thermodynamic and kinetic estimates

The Python package PyEMMA was employed to estimate the thermodynamics and kinetics of morphine binding to MOR[18]. The discrete transition-based reweighting analysis method (dTRAM) was used to construct a Markov state model by integrating US simulations and unbiased simulations[19]. Markov state models have emerged as a powerful framework for analyzing MD simulations to gain valuable insights into the underlying thermodynamics and kinetics[20, 21]. In this work, the trajectories from both the US and unbiased simulations, a total simulation time of 3.131 μs, were discretized into 101 microstates based on the reaction coordinate defined in the US simulations. Subsequently, the discretized trajectories were used to obtain a maximum-likelihood estimation of the Markov state model transition matrix in a lag time with dTRAM.

The timescales (mean-first passage times, MFPTs) of the ligand to bind to (*t_in_*) and dissociate from (*t_out_*) the binding site of MOR were identified by grouping all the microstates into two macro-states with the robust Perron Cluster Analysis (PCCA+) algorithm[22]. The kinetic constants of *k_on_* and *k_off_*, the rate constants of association and dissociation of morphine to MOR, respectively, are calculated as:

$k_{on}=\frac{1}{t_{in}*C}$ (1)

$k_{off}=\frac{1}{t_{out}}$ (2)

and the concentration of morphine *C* in this system was 0.70 mM.

The error estimations for both free energy and transition times were calculated by a bootstrap error analysis procedure[1, 21].


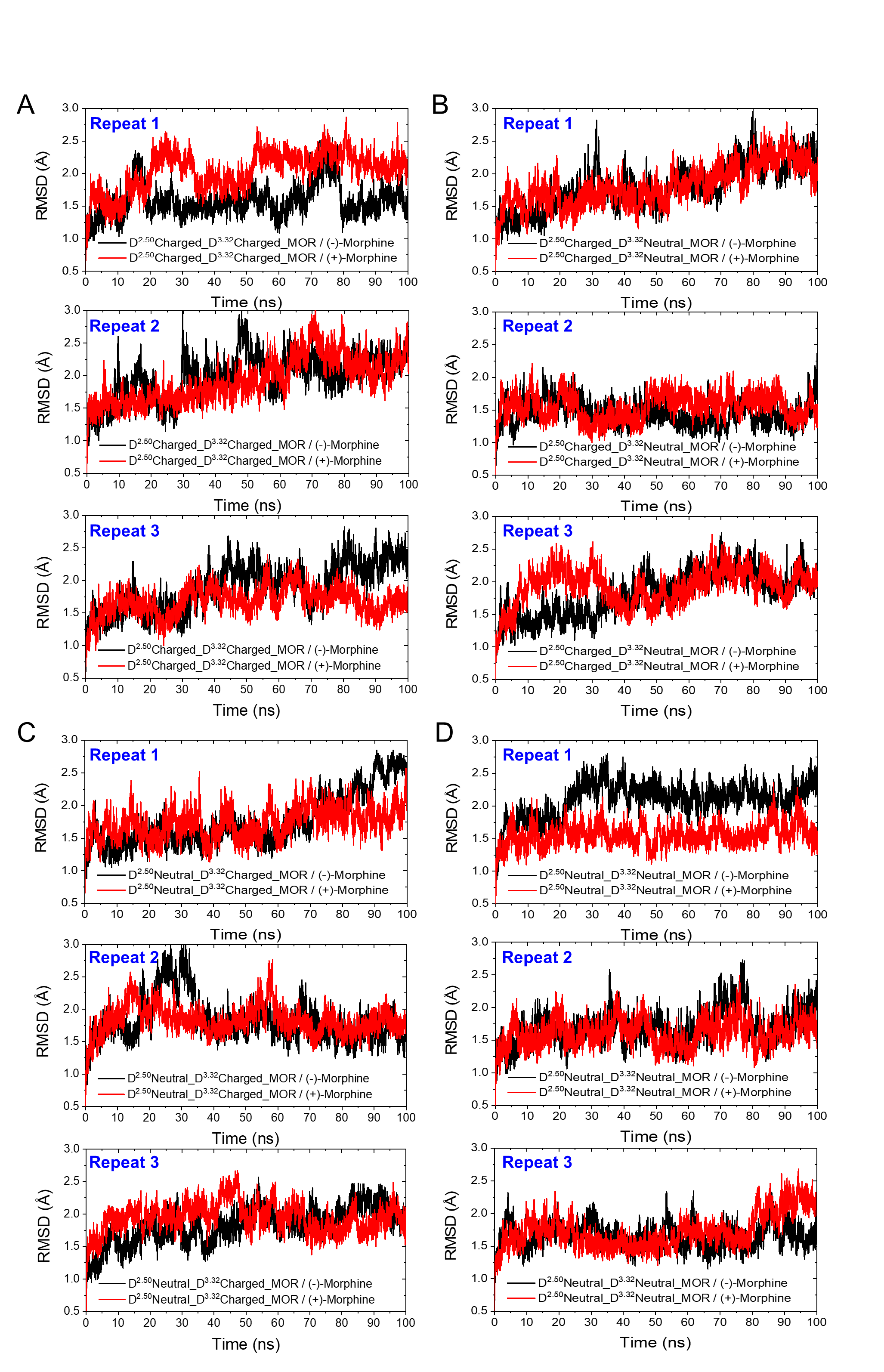
**Supplementary Figure 1.** Root-mean-square deviations (RMSDs) of backbone atoms of MORs with three parallel repeats. (A) D^2.50^Charged_D^3.32^Charged_MOR with (-)-morphine (black) or (+)-morphine (red); (B) D^2.50^Charged_D^3.32^Neutral_MOR with (-)-morphine (black) or (+)-morphine (red); (C) D^2.50^Neutral_D^3.32^Charged_MOR with (-)-morphine (black) or (+)-morphine (red); (D) D^2.50^Neutral_D^3.32^Neutral_MOR with (-)-morphine (black) or (+)-morphine (red).

**
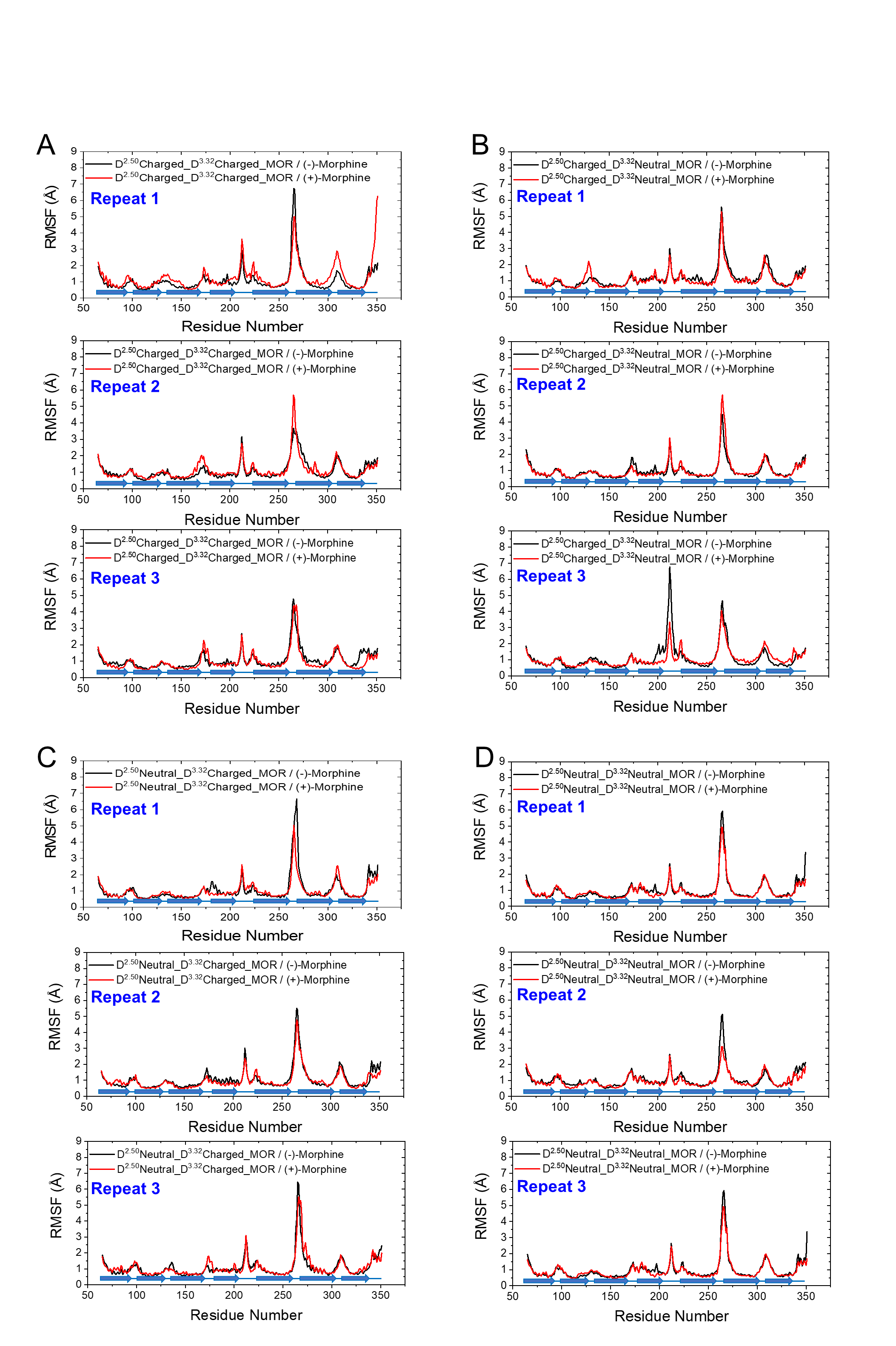
Supplementary Figure 2.** Root-mean-square fluctuations (RMSFs) of the Cα atoms of MORs with three parallel repeats. (A) D^2.50^Charged_D^3.32^Charged_MOR with (-)-morphine (black) or (+)-morphine (red); (B) D^2.50^Charged_D^3.32^Neutral_MOR with (-)-morphine (black) or (+)-morphine (red); (C) D^2.50^Neutral_D^3.32^Charged_MOR with (-)-morphine (black) or (+)-morphine (red); (D) D^2.50^Neutral_D^3.32^Neutral_MOR with (-)-morphine (black) or (+)-morphine (red).

**
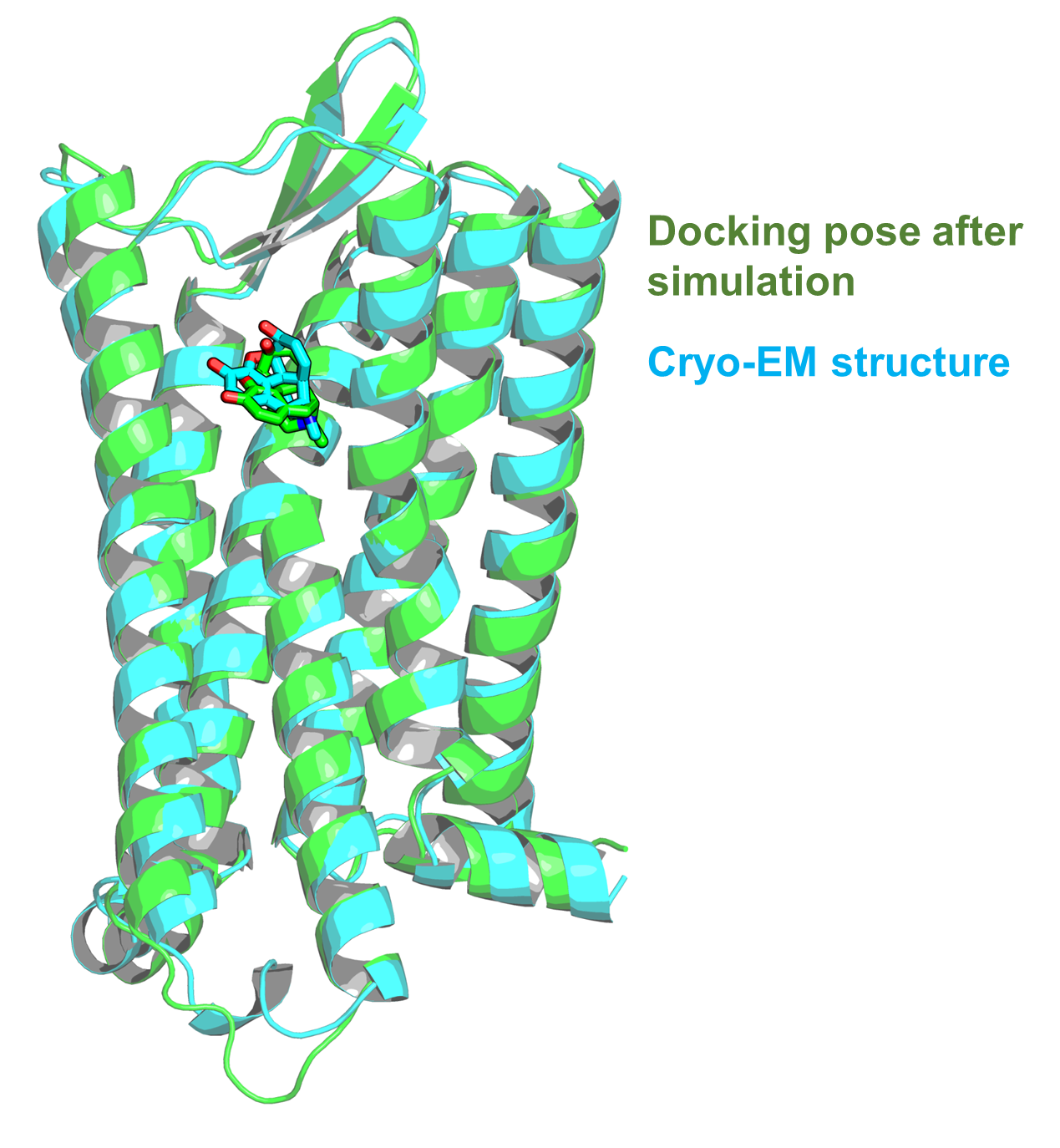
**

**Supplementary Figure 3.** Alignment of the equilibrated docking pose (green) with the crystal structure of morphine-bound MOR (PDB ID: 8EF6, in cyan). For clarity of the figure, a portion of TM6 has been omitted.

**
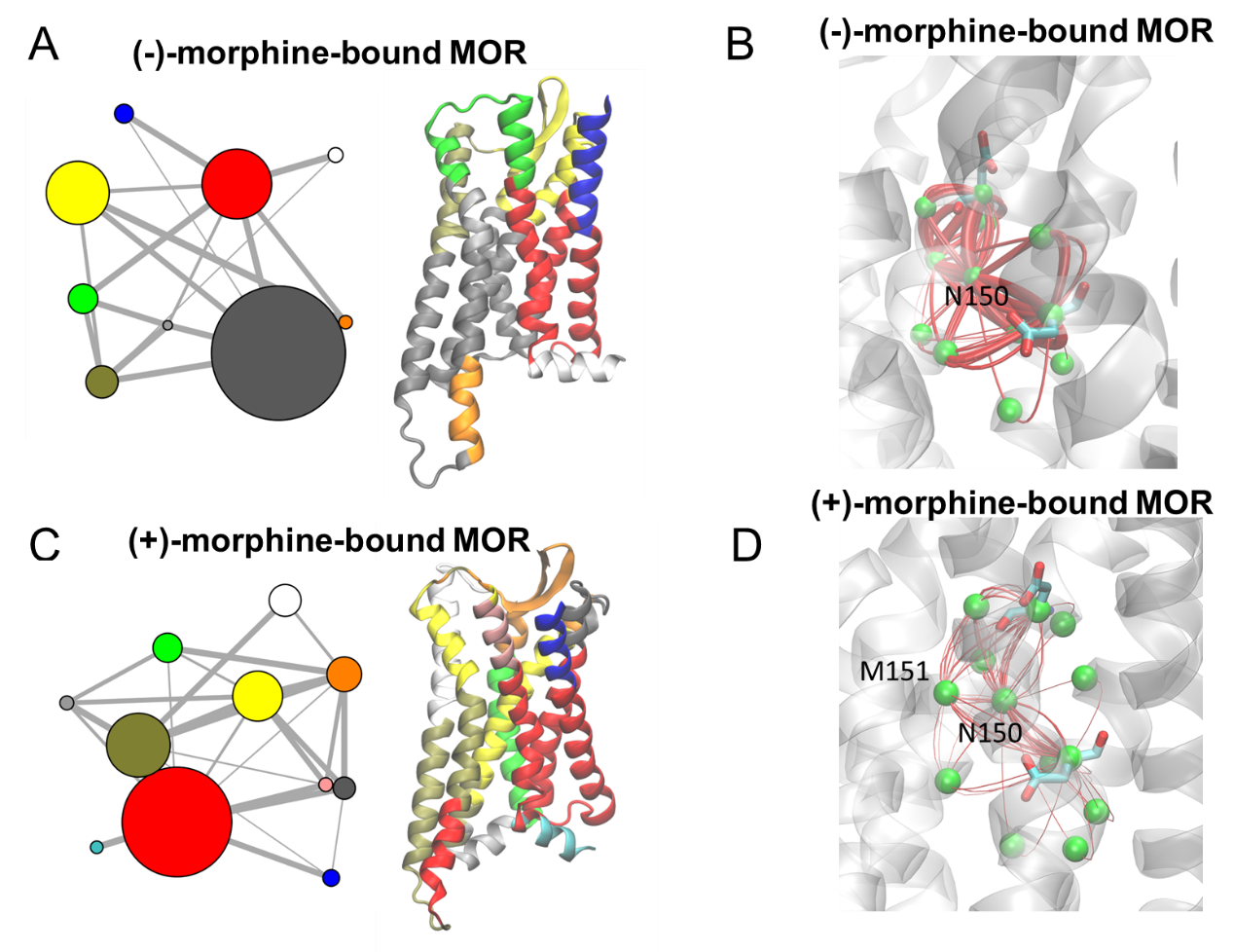
**

**Supplementary Figure 4.** Residue communication network analysis. (A) Community partitioning from correlation network analysis for (-)-morphine-bound D^2.50^Charged_D^3.32^Charged_MOR. The colors in the cartoon of MOR match the community partitioning. (B) Optimal and suboptimal path analysis for the D114 and D147 pair in the (-)-morphine-bound D^2.50^Charged_D^3.32^Charged_MOR. (C) Community partitioning from correlation network analysis for (+)-morphine-bound D^2.50^Charged_D^3.32^Charged_MOR. The colors in the cartoon of MOR match the community partitioning. (D) Optimal and suboptimal path analysis for the D114 and D147 pair in (+)-morphine-bound D^2.50^Charged_D^3.32^Charged_MOR.

**
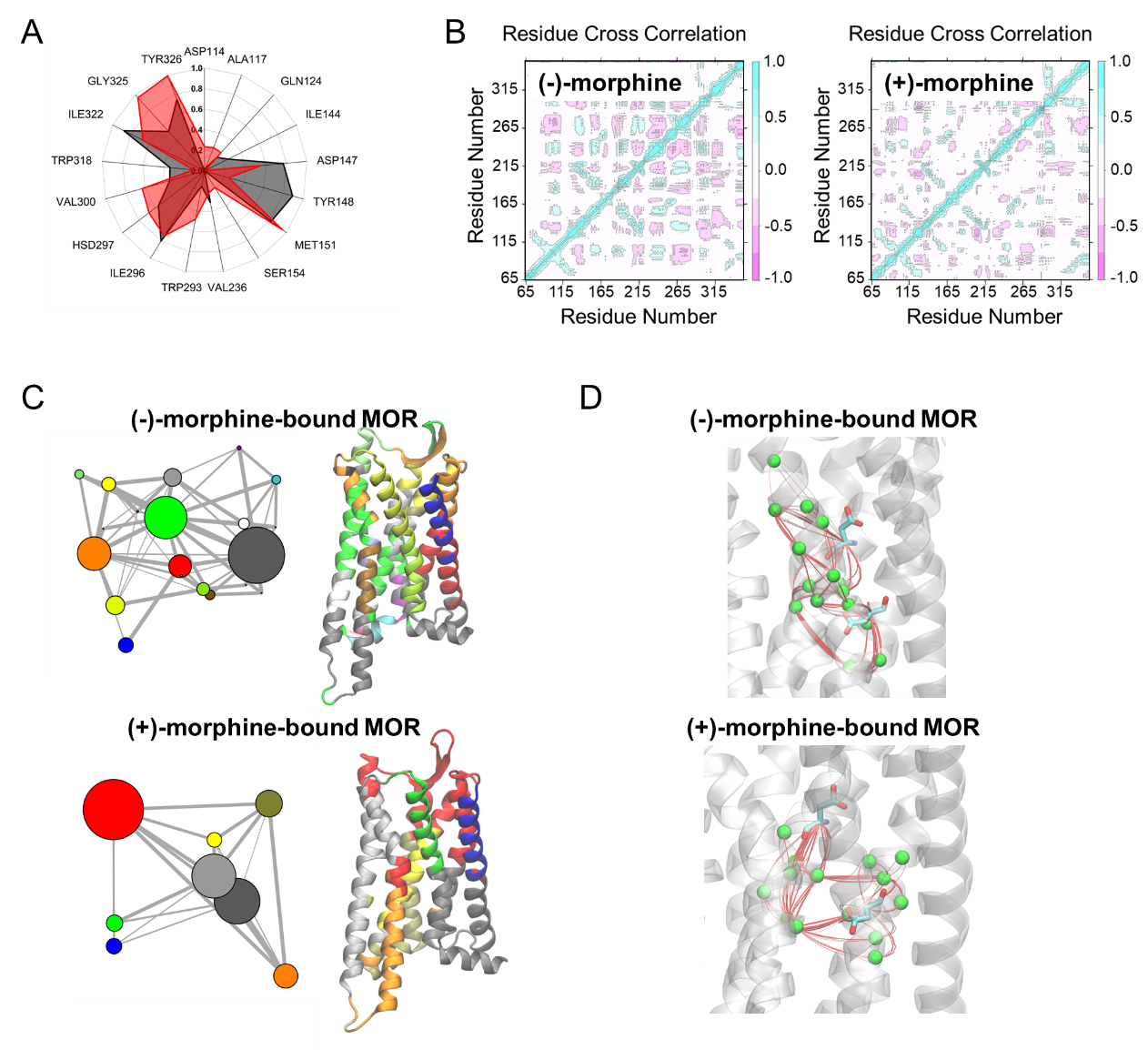
**

**Supplementary Figure 5. Trajectory analysis for D^2.50^Charged_D^3.32^Neutral_MOR.** (A) Molecular interaction fingerprints between MOR and (-)-morphine/(+)-morphine based on the last 50 ns simulations. The numbers 0-1.0 in the radar chart indicate the probability of interactions between morphine and a certain residue based on 5000 snapshots extracted from the last 50 ns simulations. The side chains of key residues interacting with morphine are displayed. The majority of interactions are hydrogen bonds and hydrophobic interactions. (B) Dynamic cross-correlation analysis of the Cα atoms in MOR during the last 50 ns of the trajectory. The color-coded representation shows the degrees of correlation and anti-correlation among the Cα atoms. The (-)-morphine-bound MOR exhibits more correlated and anti-correlated conformational motions compared to the (+)-morphine-bound MOR. (C) Community partitioning from correlation network analysis for morphine-bound MOR. The colors in the cartoon representation of MOR correspond to the community partitioning. There are more nodes in the (-)-morphine-bound MOR compared to the (+)-morphine-bound MOR, suggesting differences in the dynamic behavior and connectivity of residues. (D) Optimal and suboptimal path analysis for the D114^2.50^ and D147^3.32^ pair in the morphine-bound MOR. No particular key residues are observed to mediate the dynamic communication between D114^2.50^ and D147^3.32^.

**
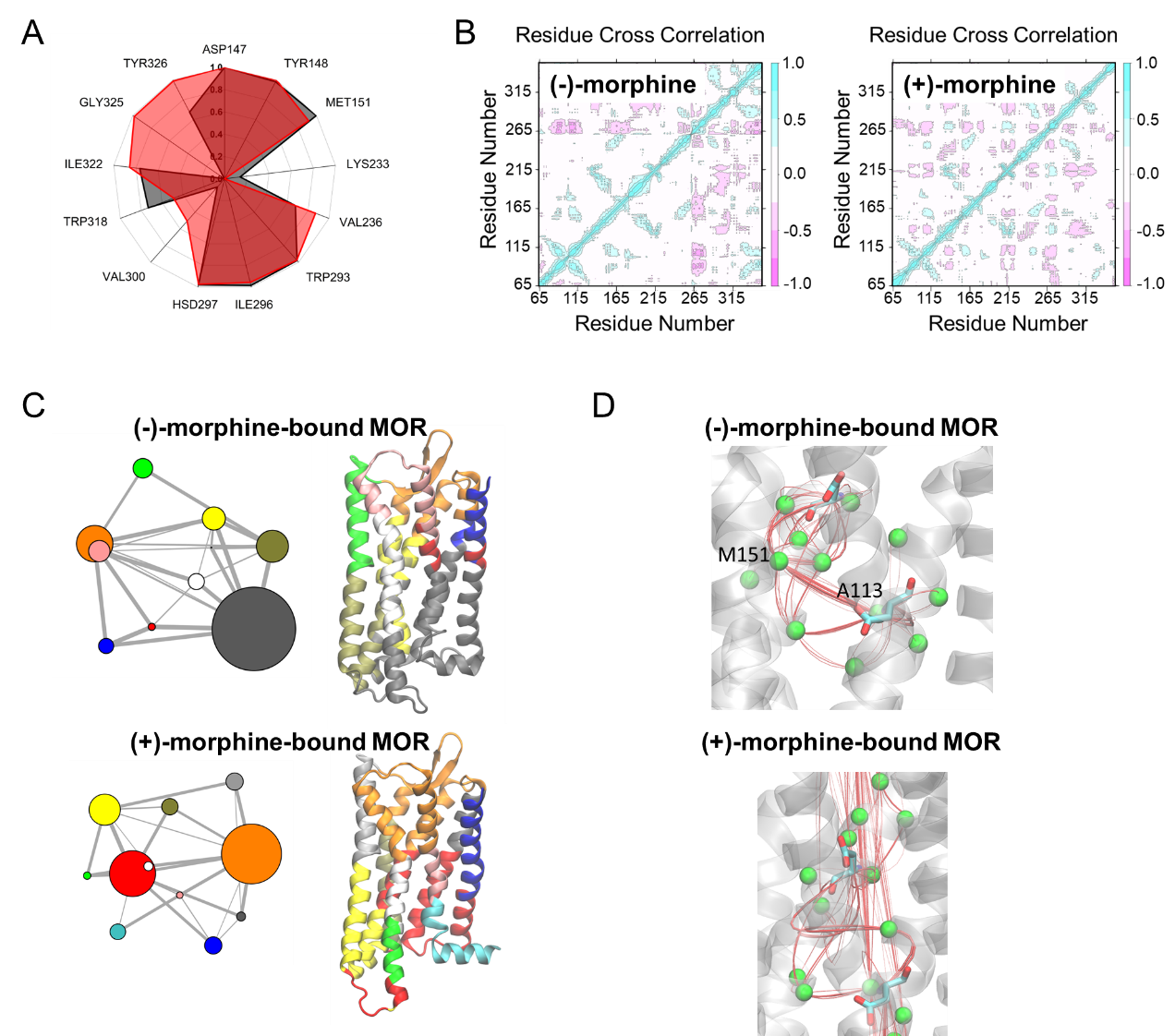
**

**Supplementary Figure 6. Trajectory analysis for D^2.50^Neutral_D^3.32^Charged_MOR.** (A) Molecular interaction fingerprints between MOR and (-)-morphine/(+)-morphine based on the last 50 ns of simulations. The numbers 0-1.0 in the radar chart indicate the probability of interactions between morphine and a certain residue based on 5000 snapshots extracted from the last 50 ns simulations. The side chains of key residues interacting with morphine are displayed. Major interactions are salt bridges, hydrogen bonds and hydrophobic interactions. Interestingly, (+)-morphine interacts with more residues than (-)-morphine. (B) Dynamic cross-correlation analysis of the Cα atoms in MOR during the last 50 ns of the trajectory. The color-coded representation shows the degrees of correlation and anti-correlation among the Cα atoms. The (-)-morphine-bound MOR shows less correlated and anti-correlated conformational motions than the (+)-morphine-bound MOR. (C) Community partitioning from correlation network analysis for morphine-bound MOR. The colors in the cartoon of MOR match the community partitioning. A similar number of nodes in the (-)-morphine-bound MOR and (+)-morphine-bound MOR was revealed. (D) Optimal and suboptimal path analysis for the D114^2.50^ and D147^3.32^ pair in the morphine-bound MOR. A113 and M151 are observed to mediate the dynamic communication between D114^2.50^ and D147^3.32^ in the (-)-morphine-bound MOR. No particular key residues are observed to mediate the dynamic communication between D114^2.50^ and D147^3.32^ in the (+)-morphine-bound MOR.

**
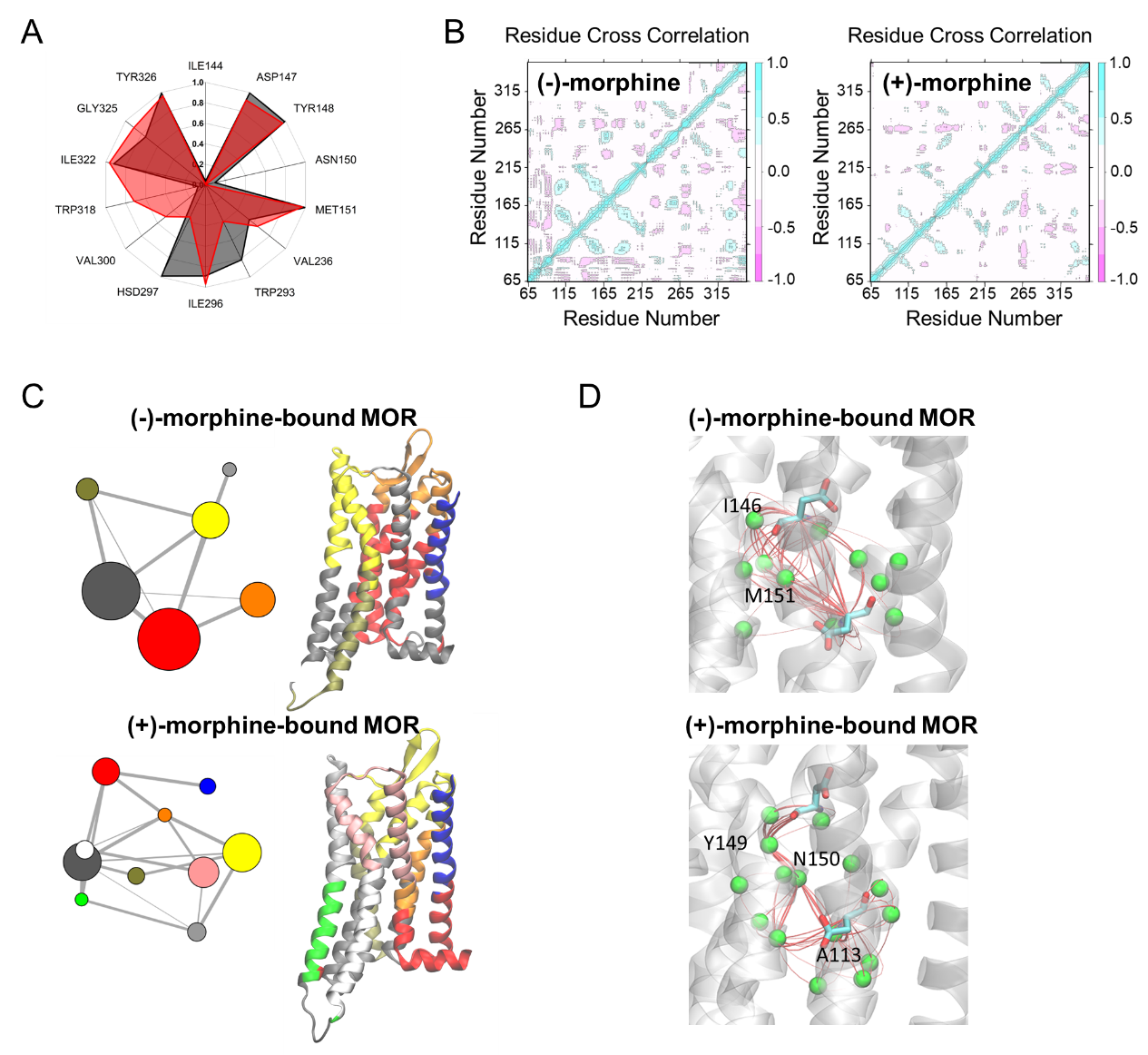
**

**Supplementary Figure 7. Trajectory analysis for D^2.50^Neutral_D^3.32^Neutral_MOR.** (A) Molecular interaction fingerprints between MOR and (-)-morphine/(+)-morphine from the last 50 ns simulations. The numerical values ranging from 0 to 1.0 in the radar chart indicate the probability of interactions between morphine and a certain residue based on 5000 snapshots extracted from the last 50 ns of the simulations. The side chains of key residues involved in interactions with morphine are visually represented. The major interactions are hydrogen bonds and hydrophobic interactions. (B) Dynamic cross-correlation analysis of the Cα atoms in MOR during the last 50 ns of the trajectory. The colors on the correlation map correspond to the degree of correlation and anti-correlation, as indicated by the color bar. The (-)-morphine-bound MOR shows more correlated and anti-correlated conformational motions compared to the (+)-morphine-bound MOR. (C) Community partitioning from correlation network analysis for morphine-bound MOR. The colors in the cartoon of MOR match the community partitioning. There are more nodes in the (+)-morphine-bound MOR than in the (-)-morphine-bound MOR. (D) Optimal and suboptimal path analysis for the D114^2.50^ and D147^3.32^ pair in the morphine-bound MOR. I146 and M151 are observed to mediate the dynamic communication between D114^2.50^ and D147^3.32^ in the (-)-morphine-bound MOR. A113, Y149, and M151 are observed to mediate the dynamic communication between D114^2.50^ and D147^3.32^ in the (+)-morphine-bound MOR.


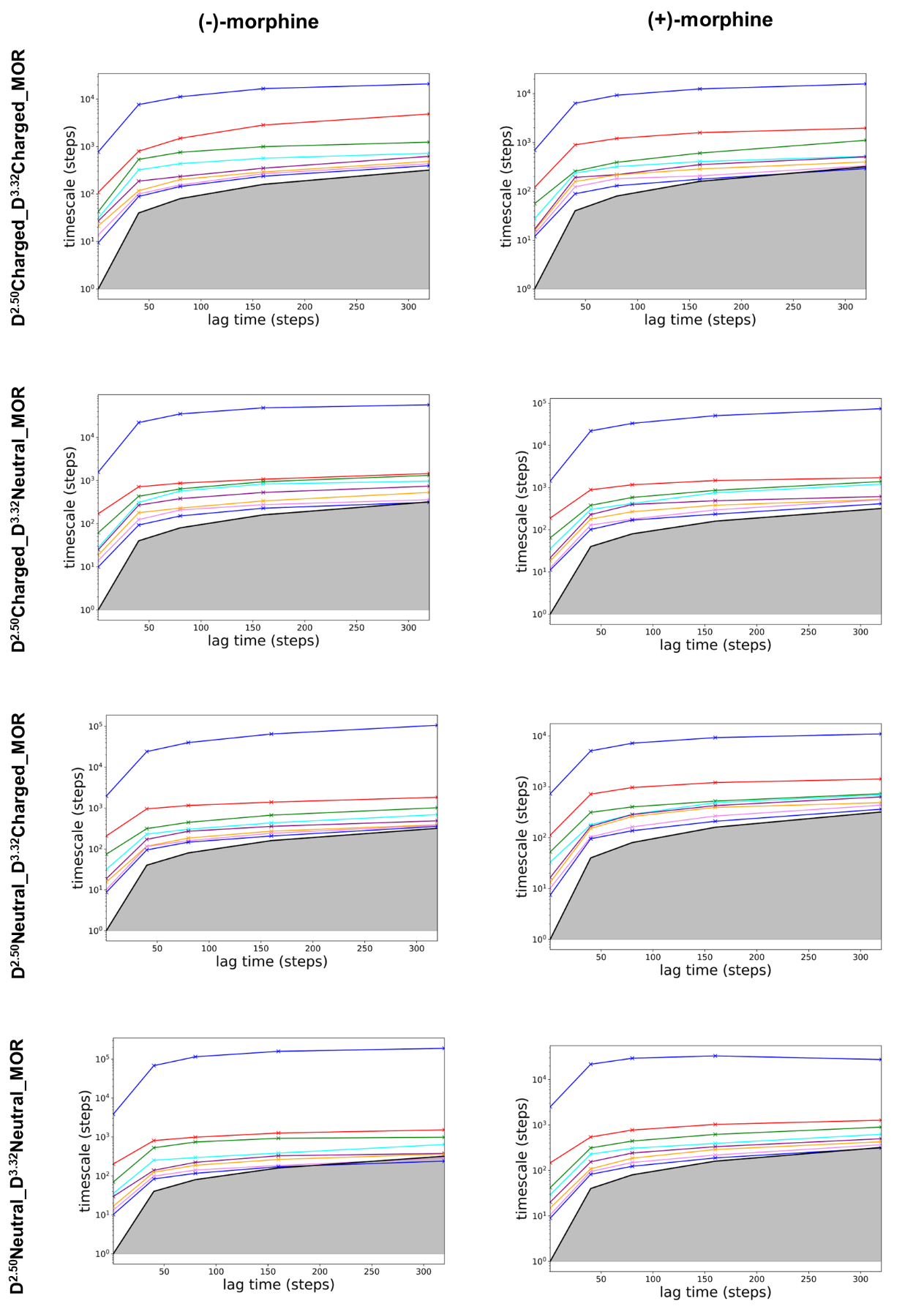


**Supplementary Figure 8.** Top eight implied timescales as a function of lag time. The Markov state model constructed at a lag time of 160 steps (1.6 ns) was used for the following analyses. The black line, along with gray shaded region, indicates the region of relaxation faster than the lag time, which the Markov state model cannot properly describe.


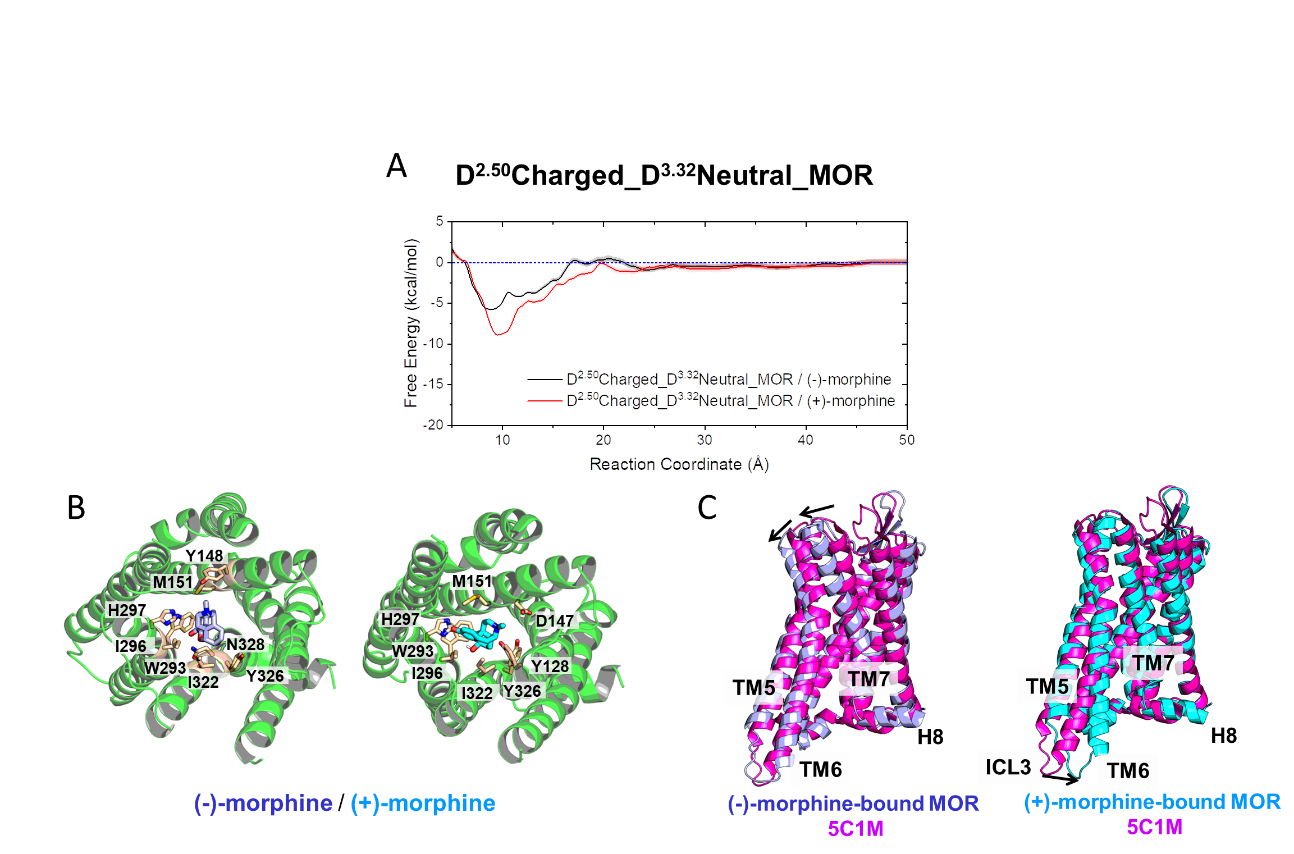


**Supplementary Figure 9.** Thermodynamic information of morphine enantiomers in D114^2.50^ deprotonated and D147^3.32^ protonated MOR. (A) Free energy profiles for the binding of (-)-morphine (black) and (+)-morphine (red) to the D114^2.50^ deprotonated and D147^3.32^ protonated MOR. The errors in the free energy estimates are obtained via a bootstrapping procedure. (B) Binding pocket residues of (-)-morphine (light blue) and (+)-morphine (cyan) in D114^2.50^ deprotonated and D147^3.32^ protonated MOR viewed from the extracellular side. (C) Alignment of (-)-morphine-bound MOR (light blue) and (+)-morphine-bound MOR (cyan) with the activated crystal structure of MOR (PDB ID: 5C1M). Arrows indicate the large displacement compared to the activated crystal structure.


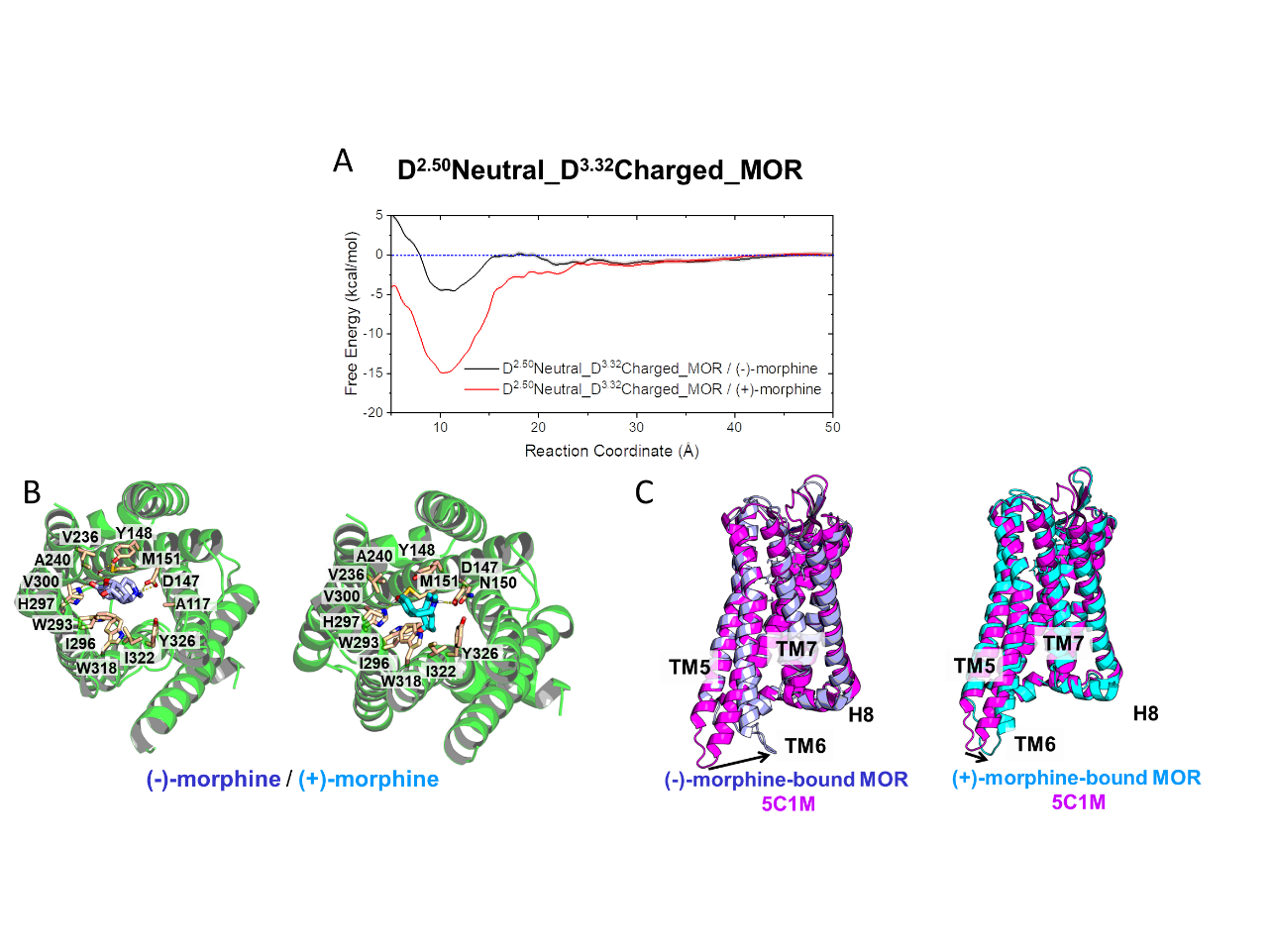


**Supplementary Figure 10.** Thermodynamic information of morphine enantiomers in D114^2.50^ protonated and D147^3.32^ deprotonated MOR. (A) Free energy profiles for the binding of (-)-morphine (black) and (+)-morphine (red) to the D114^2.50^ protonated and D147^3.32^ deprotonated MOR. The errors of the free energy are obtained via a bootstrapping procedure. (B) Binding pocket residues of (-)-morphine (light blue) and (+)-morphine (cyan) in D114^2.50^ protonated and D147^3.32^ deprotonated MOR viewed from the extracellular side. (C) Alignment of (-)-morphine-bound MOR (light blue) and (+)-morphine-bound MOR (cyan) with the activated crystal structure of MOR (PDB ID: 5C1M). Arrows indicate the large displacement compared to the activated crystal structure.


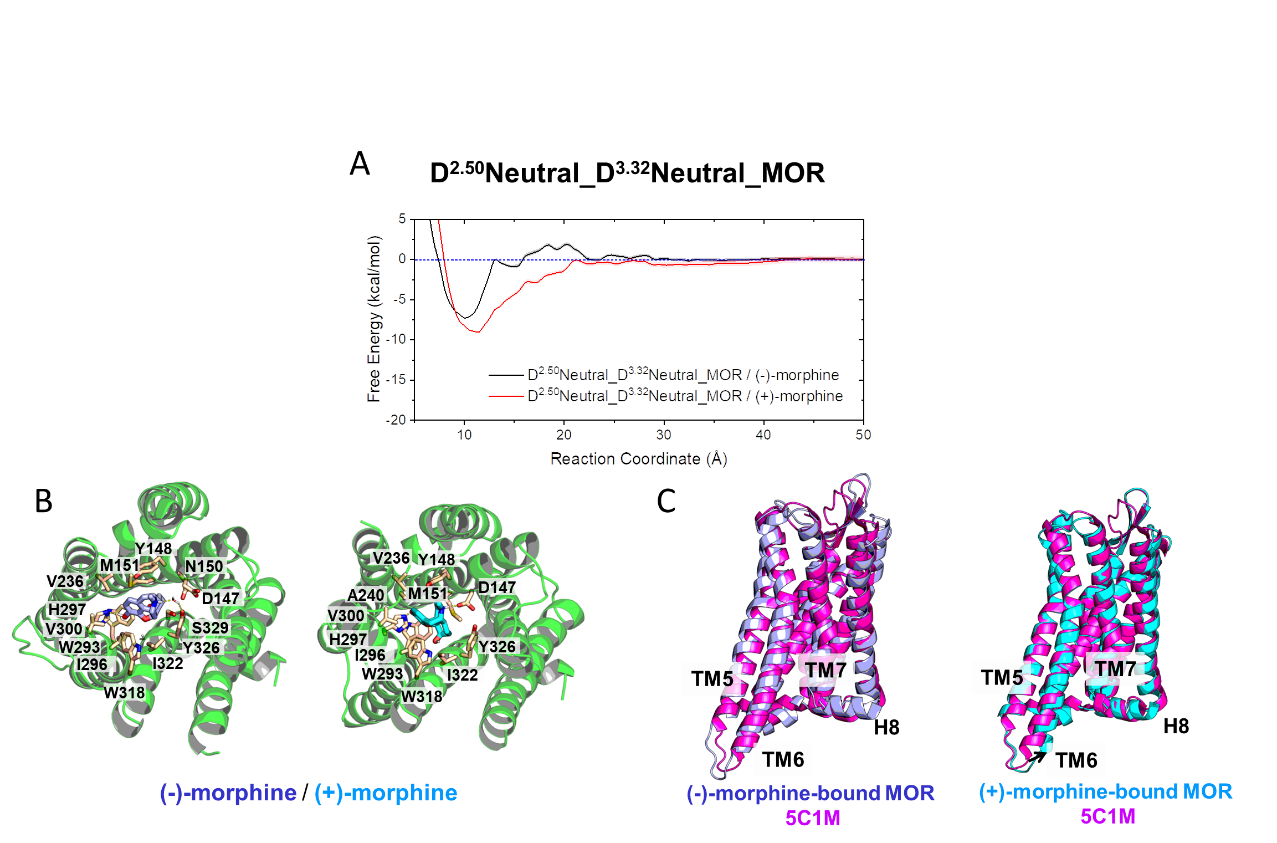


**Supplementary Figure 11.** Thermodynamic information of morphine enantiomers in D114^2.50^ and D147^3.32^ protonated MOR. (A) Free energy profiles for the binding of (-)-morphine (black) and (+)-morphine (red) to the D114^2.50^ and D147^3.32^ protonated MOR. The errors of the free energy are obtained via a bootstrapping procedure. (B) Binding pocket residues of (-)-morphine (light blue) and (+)-morphine (cyan) in D114^2.50^ and D147^3.32^ protonated MOR viewed from the extracellular side. (C) Alignment of (-)-morphine-bound MOR (light blue) and (+)-morphine-bound MOR (cyan) with the activated crystal structure of MOR (PDB ID: 5C1M). Arrows indicate the large displacement compared to the activated crystal structure is shown by arrows.

**Supplementary Table 1.** Summary of the simulations performed in this work.


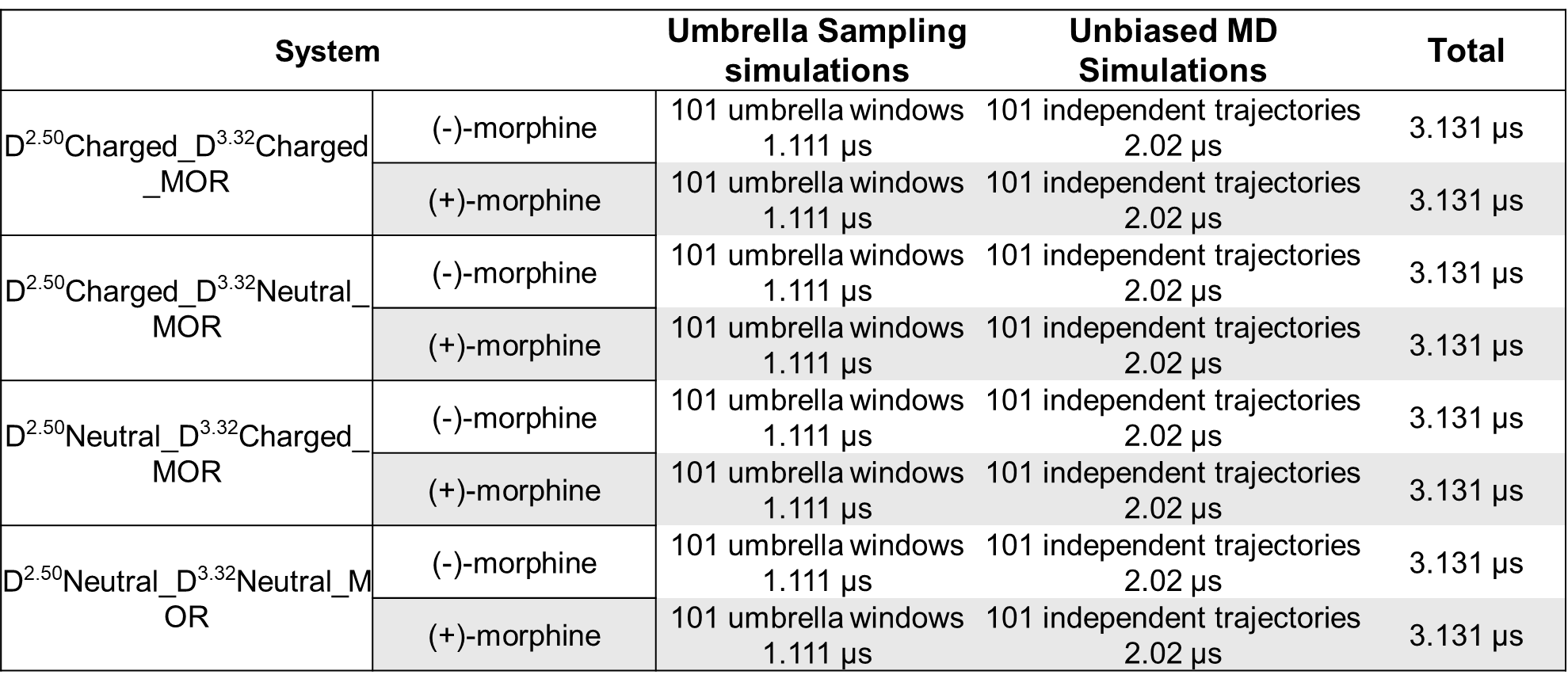


References

1. Hu, X, Wang, Y, Hunkele, A*, et al.* Kinetic and thermodynamic insights into sodium ion translocation through the mu-opioid receptor from molecular dynamics and machine learning analysis. *Plos Comput Biol*. 2019; **15**(1): e1006689.

2. Trott O, OA. AutoDock Vina: improving the speed and accuracy of docking with a new scoring function, efficient optimization and multithreading. *J Comput Chem*. 2010; **31**: 455-461.

3. Jo, S, Kim, T, Iyer, VG*, et al.* CHARMM-GUI: a web-based graphical user interface for CHARMM. *J Comput Chem*. 2008; ***29***(11): 1859-1865.

4. Best, RB, Zhu, X, Shim, J*, et al.* Optimization of the additive CHARMM all-atom protein force field targeting improved sampling of the backbone phi, psi and side-chain chi(1) and chi(2) dihedral angles. *J Chem Theory Comput*. 2012; ***8***(9): 3257-3273.

5. Huang, J, Rauscher, S, Nawrocki, G*, et al.* CHARMM36m: an improved force field for folded and intrinsically disordered proteins. *Nat Methods*. 2017; ***14***(1): 71-73.

6. Huang, L, Roux, B. Automated Force Field Parameterization for Non-Polarizable and Polarizable Atomic Models Based on Ab Initio Target Data. *J Chem Theory Comput*. 2013; **9**(8).

7. Phillips, JC, Braun, R, Wang, W*, et al.* Scalable molecular dynamics with NAMD. *J Comput Chem*. 2005; ***26***(16): 1781-1802.

8. Martyna, GJ, Tobias, DJ, Klein, ML. Constant-Pressure Molecular-Dynamics Algorithms. *J Chem Phys*. 1994; ***101***(5): 4177-4189.

9. Feller, SE, Zhang, YH, Pastor, RW*, et al.* Constant-Pressure Molecular-Dynamics Simulation - the Langevin Piston Method. *J Chem Phys*. 1995; ***103***(11): 4613-4621.

10. Essmann, U, Perera, L, Berkowitz, ML*, et al.* A Smooth Particle Mesh Ewald Method. *J Chem Phys*. 1995; ***103***(19): 8577-8593.

11. PyMol. The PyMOL Molecular Graphics System. *Schrödinger, LLC*. ***Version 2.5.0a0***.

12. Humphrey, W, Dalke, A, Schulten, K. VMD: visual molecular dynamics. *J Mol Graph*. 1996; ***14***(1): 33-38, 27-38.

13. Bouysset, C, Fiorucci, S. ProLIF: a library to encode molecular interactions as fingerprints. *J Cheminformatics*. 2021; **13**(1): 72.

14. Grant, BJ, Rodrigues, APC, ElSawy, KM*, et al.* Bio3d: an R package for the comparative analysis of protein structures. *Bioinformatics*. 2006; **22**(21): 2695-2696.

15. Yao, XQ, Malik, RU, Griggs, NW*, et al.* Dynamic Coupling and Allosteric Networks in the alpha Subunit of Heterotrimeric G Proteins. *J Biol Chem*. 2016; **291**(9): 4742-4753.

16. Scarabelli, G, Grant, BJ. Kinesin-5 allosteric inhibitors uncouple the dynamics of nucleotide, microtubule, and neck-linker binding sites. *Biophys J*. 2014; **107**(9): 2204-2213.

17. Van Wart, AT, Durrant, J, Votapka, L*, et al.* Weighted Implementation of Suboptimal Paths (WISP): An Optimized Algorithm and Tool for Dynamical Network Analysis. *J Chem Theory Comput*. 2014; **10**(2): 511-517.

18. Scherer, MK, Trendelkamp-Schroer, B, Paul, F*, et al.* PyEMMA 2: A Software Package for Estimation, Validation, and Analysis of Markov Models. *J Chem Theory Comput*. 2015; **11**(11): 5525-5542.

19. Wu, H, Mey, AS, Rosta, E*, et al.* Statistically optimal analysis of state-discretized trajectory data from multiple thermodynamic states. *J Chem Phys*. 2014; **141**(21): 214106.

20. Husic, BE, Pande, VS. Markov State Models: From an Art to a Science. *J Am Chem Soc*. 2018; **140**(7): 2386-2396.

21. Wu, H, Paul, F, Wehmeyer, C*, et al.* Multiensemble Markov models of molecular thermodynamics and kinetics. *Proc Natl Acad Sci U S A*. 2016; **113**(23): E3221-3230.

22. Roblitz, S, Weber, M. Fuzzy spectral clustering by PCCA plus : application to Markov state models and data classification. *Adv Data Anal Classi*. 2013; **7**(2): 147-179.
